# Current trends in radiocarbon in Skagerrak and Kattegat assessed by brown algae from Swedish coastal waters

**DOI:** 10.1093/rpd/ncaf032

**Published:** 2025-08-28

**Authors:** K Eriksson Stenström, S Mattsson

**Affiliations:** Particle and Nuclear Physics, Department of Physics, Lund University, Professorsgatan 1, SE-223 63 Lund, Sweden; Medical Radiation Physics Malmö, Department of Translational Medicine, Lund University Skåne University Hospital Malmö, SUS Malmö, Inga Maria Nilssons gata 47, SE-205 02 Malmö, Sweden

## Abstract

Carbon-14 often dominates the effective dose to the public from authorized discharges from Swedish nuclear power plants (NPPs). In contrast to air-borne releases, water-borne discharges of ^14^C are currently not routinely monitored at Swedish NPPs. We have measured ^14^C in *Fucus* spp. (brown algae, used as bioindicators of ^14^C) in shallow waters at the Swedish west coast from 2020 to 2024. At Ringhals NPP, ^14^C in *Fucus* spp. was up to ~50 per cent higher than at nearby marine reference sites and was also higher than observed in the nearby terrestrial environment. The local marine environment of Ringhals NPP showed high spatial and temporal variability in ^14^C. Carbon-14 in *Fucus* spp. was generally higher in Skagerrak than in the more southernly Kattegat, likely mainly due to influence from discharges from the spent nuclear fuel reprocessing plant in La Hague in France and from its counterpart in Sellafield in the UK.

## Introduction

The long-lived pure beta-emitter ^14^C (*T*_1/2_ = 5730 years) is an important radionuclide produced and released from nuclear power plants (NPPs) during normal reactor operation. Its significance is not only due to the long physical half-life, but also to its high bioavailability and its role in the global carbon cycle. Carbon-14 often dominates the radiation dose to the public from NPPs, mainly from air-borne releases [1–3]. At Swedish NPPs, radioactive releases to air and water-borne discharges to the marine environment are monitored for a number of relevant radionuclides to demonstrate compliance with regulatory dose limits. However, for ^14^C, only air-borne releases, and not water-borne discharges, are currently monitored. This strategy has so far been based on the general assumption that ^14^C emissions from nuclear power reactors are mainly air-borne and that only a minor fraction is released to the water recipient [4, 5].

Brown algae (*Fucus* spp.) are commonly used as bioindicators of marine radioactive pollution in environmental monitoring programmes at Swedish NPPs. Despite a negligible contribution to the diet of the Swedish population, *Fucus* spp. are suitable as bioindicator organisms, since they are sessile (permanently attached to the seabed), abundant and have high concentration factors for many radionuclides of relevance for the nuclear power industry. Carbon isotopes, in the form of dissolved inorganic carbon (DIC) in seawater, enter *Fucus* spp. through photosynthesis.

We have recently demonstrated the presence of a significant environmental signal of ^14^C in *Fucus* spp. collected in spring 2020 in the local marine environment of the Ringhals NPP (57.26 N, 12.11E) on the Swedish west coast, stemming from liquid discharges from the NPP [6]. The excess of ^14^C in *Fucus* spp., collected in shallow waters close to Ringhals NPP, was up to ~25 per cent compared to relevant nearby reference sites (sites considered not significantly affected by the NPP) [6]. This is a higher excess than what is commonly found in the terrestrial environment of Swedish NPPs (usually <10 per cent excess compared to a relevant reference site, see Eriksson Stenström and Mattsson [6] and references therein).

**Figure 1:**
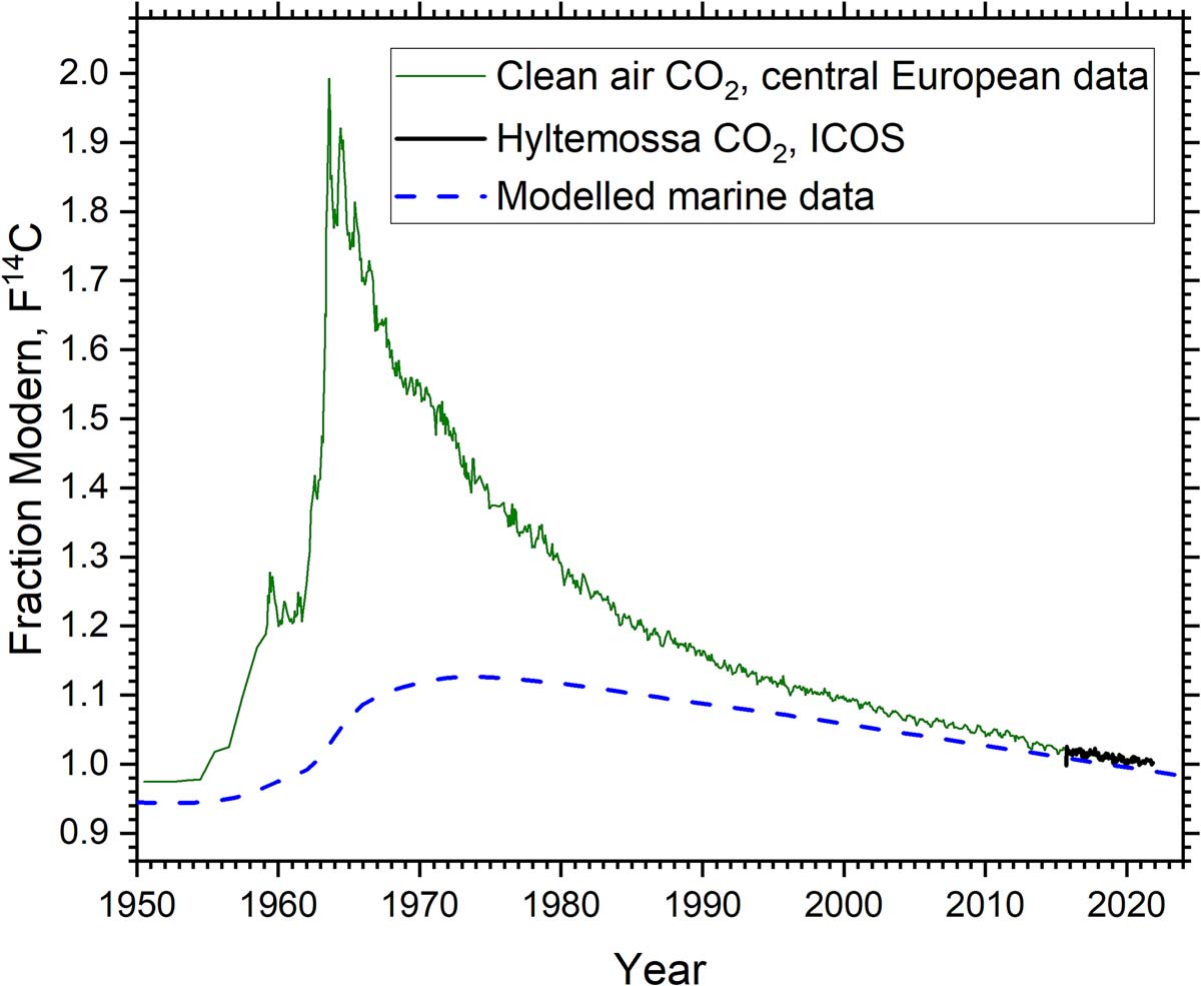
^14^C in atmospheric CO_2_ in the Northern Hemisphere (atmospheric bomb pulse) and oceans (marine bomb pulse) [7], expressed in units of fraction modern (F^14^C) [8]. F^14^C = 1.0 corresponds to ~226 Bq (kg C)^−1^. Carbon-14 data in atmospheric CO_2_ has been collected at rural background stations in Central Europe [9–12], and at the Swedish Integrated Carbon Observation System (ICOS) station Hyltemossa [13]. The dashed curve shows the modelled global marine surface mixed-layer bomb pulse for the period 1950–96 [7], and values after this were extrapolated based on linear regression of marine data from 1987 to 1996. The homogenous distribution of bomb-^14^C in the lower atmosphere results only in minor spatial variations in the atmospheric CO_2_ bomb pulse in the Northern Hemisphere, whereas the marine bomb pulse may have major variations spatially as well as in depth.

For source apportionment studies of ^14^C, which has several origins, natural as well as anthropogenic, the choice of appropriate references sites is of utmost importance. For ^14^C, there are important differences between the terrestrial and the marine environment. Reference sites in the terrestrial environment mainly have two sources of ^14^C: cosmic-ray-induced natural ^14^C and ^14^C produced from the atmospheric testing of nuclear weapons in the mid-1900s (see e.g. Eriksson Stenström and Mattsson [6]). As seen in Fig. 1, this testing almost doubled the concentration of ^14^C in atmospheric CO_2_ in a uniform manner the Northern Hemisphere by 1963 (a similar but not as high so-called bomb-pulse was seen in the Southern Hemisphere). As the natural as well as the bomb ^14^C components are in the form of carbon dioxide, ^14^C readily enters the biosphere with photosynthetic absorption into plants as the first step. Small local variations in ^14^C, relative the stable carbon isotopes ^12^C and ^13^C, in terrestrial biota can result from dilution due to combustion of fossil fuels (free of ^14^C), or due to anthropogenic ^14^C from NPPs and from industrial and laboratory uses of ^14^C.

The decrease in the atmospheric ^14^C bomb-pulse since 1963 is mainly due to the absorption of CO_2_ into the oceans and hydrosphere, but also due to uptake in the biosphere. Dilution due to large-scale combustion of fossil fuels also contributes to the decreasing ^14^C concentration in atmospheric CO_2_ seen in Fig. 1. An obvious trend in Fig. 1 is that the slope of the atmospheric ^14^C curve continues to decrease.

The choice of marine reference sites in environmental monitoring programmes at nuclear facilities must consider that ^14^C is less homogenously distributed in the marine environment than in terrestrial living biota. Natural as well as bomb-^14^C in CO_2_ enter the ocean at the surface, becoming DIC, which is distributed in the ocean depending on the rates of vertical circulation and mixing. Hence, deep waters are generally containing older DIC (depleted in ^14^C due to its radioactive decay) than surface waters. The modelled marine data in Fig. 1 represents the global average of ^14^C in the oceans down to a depth of 75 m [7], and surface waters are expected to have a concentration of ^14^C in DIC closer to the atmospheric curve than to the modelled marine curve. Furthermore, in coastal surface waters, upwelling of old water and inflow of freshwater and groundwater affect the ^14^C content of DIC [14]. These factors, including that river runoff may contain ^14^C-depleted DIC from carbonate-bearing bedrock [14], may result in considerable spatial and temporal variations in coastal waters of relevance for environmental studies of NPPs. In the study by Eriksson Stenström and Mattsson [6] and in Mattsson et al. [15], we demonstrated such variations for Swedish coastal waters for samples of *Fucus* spp. collected in spring 2020.

We have also demonstrated that Swedish west-coast waters are influenced by radioactive discharges from the spent nuclear fuel reprocessing plants La Hague in France and Sellafield in the UK [6, 15]. These studies have used a unique biobank, containing *Fucus* spp. samples regularly collected at the site Särdal (56.76 N, 12.63E) since 1967. The temporal variations have been studied for several radionuclides, including ^14^C [15]. For ^14^C, a pronounced rise around the turn of the millennium correlates with increased discharges from the reprocessing facilities, taking site-specific transport times and dilution factors into account [6, 15].

One of the aims of this paper is to further demonstrate the importance of choice of proper reference sites in the marine environment for environmental studies of ^14^C, by additional investigations of temporal and spatial variations in Swedish coastal waters, focussing on the west coast. Another aim is continued studies of the spatial and temporal impact of water-borne DIC discharges from Ringhals NPP on the local marine environment of the NPP, by means of analysis of ^14^C in *Fucus* spp. used as bioindicators of ^14^C. We also demonstrate that the spatial and temporal variations in ^14^C are higher in biota in Swedish coastal waters than in the terrestrial biosphere.

## Materials and methods

### Sampling sites

**Figure 2:**
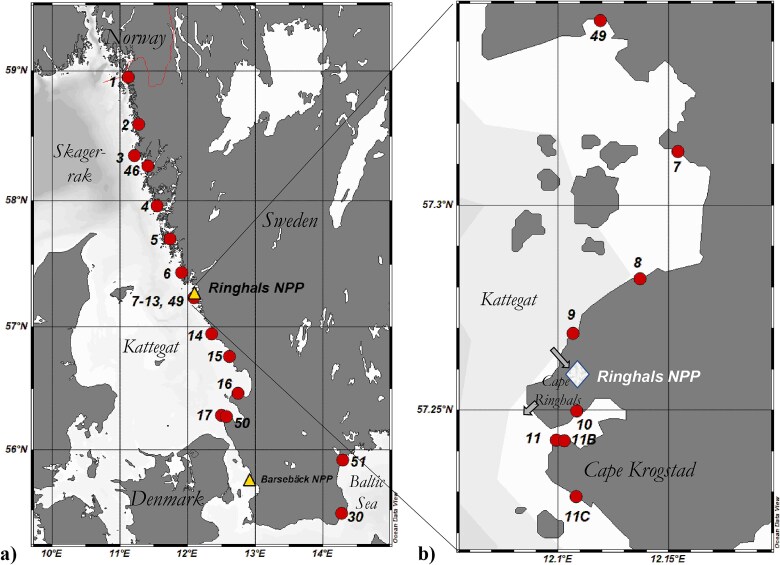
(a) Map indicating the relevant sampling sites in the current and previous study [6]. (b) Enlargement showing the sampling sites around Ringhals NPP. The arrows indicate the inlet and outlet of cooling water. The latter carries water-borne radioactive discharges from the NPP. The cooling water is discharged towards the south, and strong water currents disperse the cooling water plume mainly towards the north. Sites 1–30 were used in the previous study [6]. Sites 1, 3, 4, 7–11, 17, and 30 were revisited in the present current study and Sites 11B, 11C, 46, 49, 50, and 51 have been added. Maps: Schlitzer, Reiner, Ocean Data View, https://odv.awi.de, 2025.

The study area is shown in Fig. 2a, which also includes the location of the operational Ringhals NPP (57.26 N, 12.11E) at Kattegat on the west coast and the decommissioned Barsebäck NPP located further south. As described previously [6], the salinity of the water on the Swedish west coast decreases significantly from north to south. In the northernmost waters of Skagerrak, the salinity can exceed 2.5 per cent, due to inflow of bottom water with high salinity from the North Sea. In the southern Baltic Sea, the salinities are less than 0.8 per cent. Surface water currents flowing out from the Baltic Sea into Kattegat, and then reaching Skagerrak, result in the gradient in salinity along the Swedish west coast. Several larger and smaller rivers also enter the waters of the Swedish west coast. Depending on the local geology, these may be depleted in ^14^C, due to dissolution of fossil carbonates from the bedrock into the river water [14].

The study area at Ringhals NPP is presented in Fig. 2b. The inlets and outlets of cooling water at Cape Ringhals, where the NPP is located, are also indicated in the figure. The outlet water, carrying waste from the reactor coolant water purification and treatment system, travels in 2 km long tunnels that enter the sea at 5–6 m beneath the water surface [16]. The water is released in a southerly direction and is mixed horizontally as well as vertically [16]. The sea water off Cape Ringhals is characterized by a large saltwater stratification, and the surface water changes direction on a frequent basis (often more than one change per day between northern and southern currents), giving rise to whirlpools north of the cape [16]. According to Notter [16], a 1-year study of the dispersion of the cooling water plume from Ringhals NPP showed that the direction was towards the north at 40 per cent of the time, towards the south at 25 per cent, and to the west at 15 per cent. The direction was undefined for the remaining 20 per cent of the time.

Prior to the start of sampling in the marine environment, one of the Ringhals reactors had just been taken out of operation (the pressurized water reactor [PWR] R2, 900 MW_e_, in operation from 1975 to the end of 2019). Another of the Ringhals reactors was shut down at the end of 2020 (the boiling water reactor [BWR] R1, 881 MW_e_, in operation 1976 to the end of 2020). Two reactors were operational during the whole sampling period, both being PWRs (R3, 1070 MW_e_, in operation since 1981; and R4, 1120 MW_e_, since 1983). The two BWRs at Barsebäck NPP (55.74 N, 12.92E; both reactors 600 MW_e_), see Fig. 2, were shut down already in 1999 and 2005, respectively, and the NPP is now under decommissioning and dismantling. The other two Swedish NPPs, Oskarshamn NPP and Forsmark NPP, are located on the Swedish east coast at the Baltic Sea, and are of less interest for the current investigation. Figure 2a and b also show the sites used for sampling of *Fucus* spp. (see Supplementary Material S1 for further details). Oskarshamn NPP is located ~300 km along the coast north of the Site 30. Skillinge and 51. Åhus, and Forsmark NPP is sited further north in the Gulf of Bothnia [6]. In the previous study of Eriksson Stenström and Mattsson [6], 4 sites in Skagerrak and 13 sites in Kattegat were used in spring 2020 for collection of *Fucus* spp. In the present study, three of the Skagerrak sites (Sites 1, 3, and 4) and six of the Kattegat sites (Sites 7–11 and 17) were revisited for additional sampling (the site numbers of these are the same as in Eriksson Stenström and Mattsson [6]). One new site was added for Skagerrak (46. Lysekil) and four new sites were added for Kattegat (49. Åsa, 11B. Bua at the old pier; 11C. Bua strand; and 50. Arild). Åsa (Site 49, 57.24 N, 12.12E), located about 10 km north of Ringhals NPP, was chosen to further cover the Ringhals area.

In our previous study (sampling in spring 2020) [6], the *Fucus* spp. collected at Site 11 (Bua, located ~1.3 km SSE of the outlet of the liquid discharges from Ringhals NPP) had the highest ^14^C concentration of all sites. The cooling water outlet, which also carries the liquid discharges from the NPP, is directed towards the south [16], which explains that the highest excess in ^14^C was seen south of the NPP [6]. The new Sites 11B and 11C (Fig. 2b) were chosen to improve the spatial resolution for further studies of the local variations in ^14^C the immediate vicinity of Ringhals NPP. The new Site 11B is located ~200 m east of Site 11, and 11C is ~1.5 km south of Site 11 at Cape Krogstad (see Fig. 2b). Sites 11 and 11B are both in the fjord Båtafjorden, whilst Site 11C faces open sea.

The new Site 50 (Arild), located in the southernmost Kattegat, was chosen as an alternative to the previous Site 17 (Mölle). Two reference sites were used for the Baltic Sea: Skillinge (Site 30, 55.47 N, 14.85E), which was also used in our previous study [6], and Åhus (Site 51, 55.91 N, 14.30E; *Fucus* was easier to sample at this site than at Skillinge). In addition, eight of the sites were selected for sampling of grass to assess ^14^C in the terrestrial environment (Sites 1, 46, 9, 10, 11, 11B, 50, and 30; see Supplementary Materials for further information).

### Sampling, sample preparation, and analysis of ^14^C

In the previous study [6], *Fucus* spp. (mainly *Fucus vesiculosus*) was collected in spring 2020 at Sites 1–17 at the Swedish west coast and at the Site 30. Skillinge in the Baltic Sea. To extend this data series, *Fucus* spp. was collected in autumn 2020 (9 sites), in autumn 2022 (14 sites), in spring 2023 (9 sites), in autumn 2023 (9 sites) and in spring 2024 (9 sites). The sampling of *Fucus* spp. and one sample of *Ascophyllum nodosum* (Site 3. Smögen, 12 October 2022) was performed in the same manner as previously described [6]. Sampling of *F. vesiculosus* was prioritized, and for some sites *F. serratus* was also collected (see Supplementary Materials). In our previous study [6], the upper part of the *F. vesiculosus* individuals were mainly analysed (denoted ‘vesicles and above’ in the Supplementary Material S2), mainly representing *Fucus* tissue formed since last spring when the vesicles (air bladders) are formed. This approach continued for samples collected until 2022 (see further details in our previous study [6]). For samples collected in 2023 and 2024, whole plants were used. This change of approach is justified in our previous study [6], where it was demonstrated that in samples, for example from Särdal (Site 15) and from the Baltic Sea reference site Skillinge (Site 30), there were no significant differences in ^14^C for the various fractions of the algae representing different periods of growth. A further support to the change in methodology is that the main mass of the algae plant consists of newly formed material, as both *F. vesiculosus* and *F. serratus* defoliate (shedding older fronds after fruiting [17]). None of these samples were however collected in the vicinity of Ringhals NPP [6]. Hence, different parts of individual algae plants were now analysed to further assess the variation in ^14^C in DIC close to the NPP. Grass was sampled eight sites at different sampling campaigns between 2020 and 2024 (see Supplementary Material S3).

For each site and sampling date, three *Fucus* spp. individuals were pooled into a single sample prior to drying at 55°C to constant weight. Sample pretreatment (including acid–alkali–acid pretreatment to remove carbonates and organic acids) and ^14^C analysis was performed at the Radiocarbon Dating Laboratory at Lund University, as described previously [6]. The results are reported as Fraction Modern, F^14^C [8]. F^14^C = 1 corresponds to a specific activity of about 228 Bq (kg C)^−1^ [18]. Analytical uncertainties are reported at one standard deviation (1*σ*).

## Results and discussion

All data are presented in the Supplementary Materials.

### General trends 2020–24

**Figure 3:**
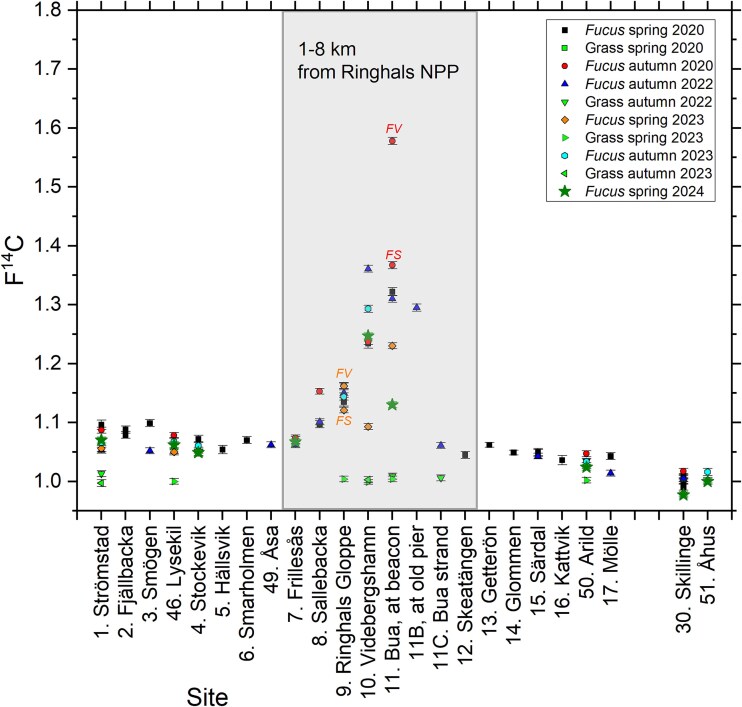
F^14^C in *Fucus* spp. and grass collected at the marine sites from 2020 to 2024. For Skagerrak and Kattegat, the sites are sorted according to longitude from north to south: Site 1 (Strömstad) is in the far north of the west coast, and Sites 17 (Mölle) and 50 (Arild) are the southernmost sites on the west coast. Sites 30 (Skillinge) and 51 (Åhus) serve as reference sites in the Baltic Sea. Data from spring 2020 is published in Eriksson Stenström and Mattsson [6], data from Särdal in Eriksson Stenström and Mattsson [6] and Mattsson et al. [15]. The uncertainty bars represent 1*σ*, resulting from repeated measurement of the same sample. *FV*: *Fucus vesiculosus*; *FS*: *Fucus serratus*.

All F^14^C data for *Fucus* spp. collected at the described marine sites along the Swedish west coast in 2020–24 are shown in Fig. 3 (including relevant data from our previous study [6]), as well as F^14^C in grass samples. It is evident that F^14^C is varying considerably in the marine environment, whereas F^14^C in the terrestrial environment does not (see further discussion below). The major trends for F^14^C in *Fucus* spp. from spring 2020, reported in our previous study [6], are confirmed for the other sampling campaigns. Firstly, for all seasons investigated, the local marine environment of Ringhals NPP (shaded area in Fig. 3) shows the highest F^14^C values, due to liquid discharges from the NPP (see below). Secondly, Skagerrak generally has higher F^14^C in *Fucus* spp. than southern Kattegat, and both Skagerrak and Kattegat show higher F^14^C than the Baltic Sea, as also reported previously [6].

### Trends outside the Ringhals nuclear power plant area

**Figure 4:**
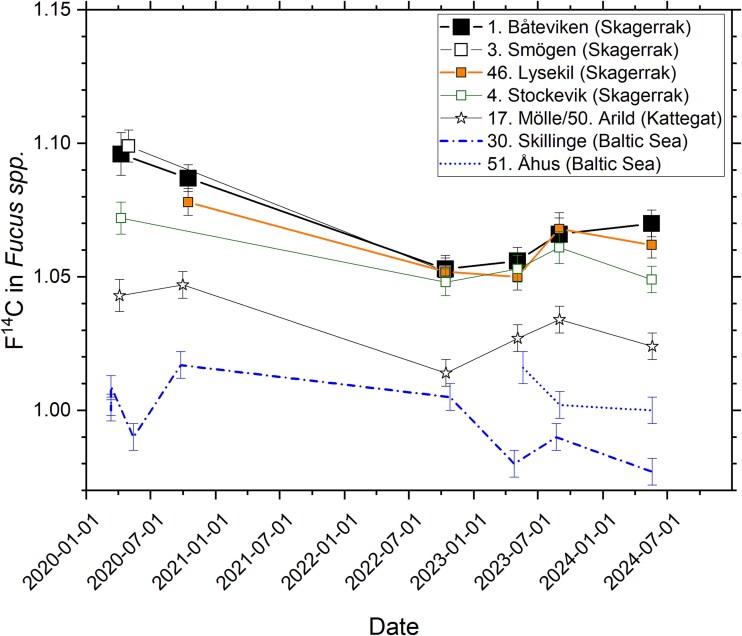
Temporal variation in F^14^C at sites in Skagerrak and Kattegat on the Swedish west coast, and at two reference sites in the Baltic Sea. The sites at Skagerrak and Kattegat are ranked from north to south in the legend. The uncertainty bars represent 1*σ*, resulting from repeated measurement of the same sample.

The temporal variation in F^14^C from 2020 to 2024 for the sites of repeated collection of *Fucus* spp. in Skagerrak and Kattegat, excluding the sites located within 10 km of Ringhals NPP, is shown in Fig. 4. Also, data for the two Baltic Sea reference sites are included, demonstrating lower F^14^C in the Baltic Sea than in Skagerrak and Kattegat (as previously reported [6]). Furthermore, F^14^C still decreases towards the south at the Swedish west coast. In contrast to the data from 2020 [6] to 2024, the data from 2022 to 2023 show no distinct increase in F^14^C with increasing latitude for Skagerrak. However, the Skagerrak F^14^C data for 2022–24 are still higher than F^14^C in *Fucus* spp. at the Kattegat site (Fig. 4). As stated earlier, the phenomenon of lower F^14^C in Kattegat than in Skagerrak can be attributed to increasing dilution of ^14^C from the La Hague and Sellafield towards the south of the Swedish west coast.

**Figure 5:**
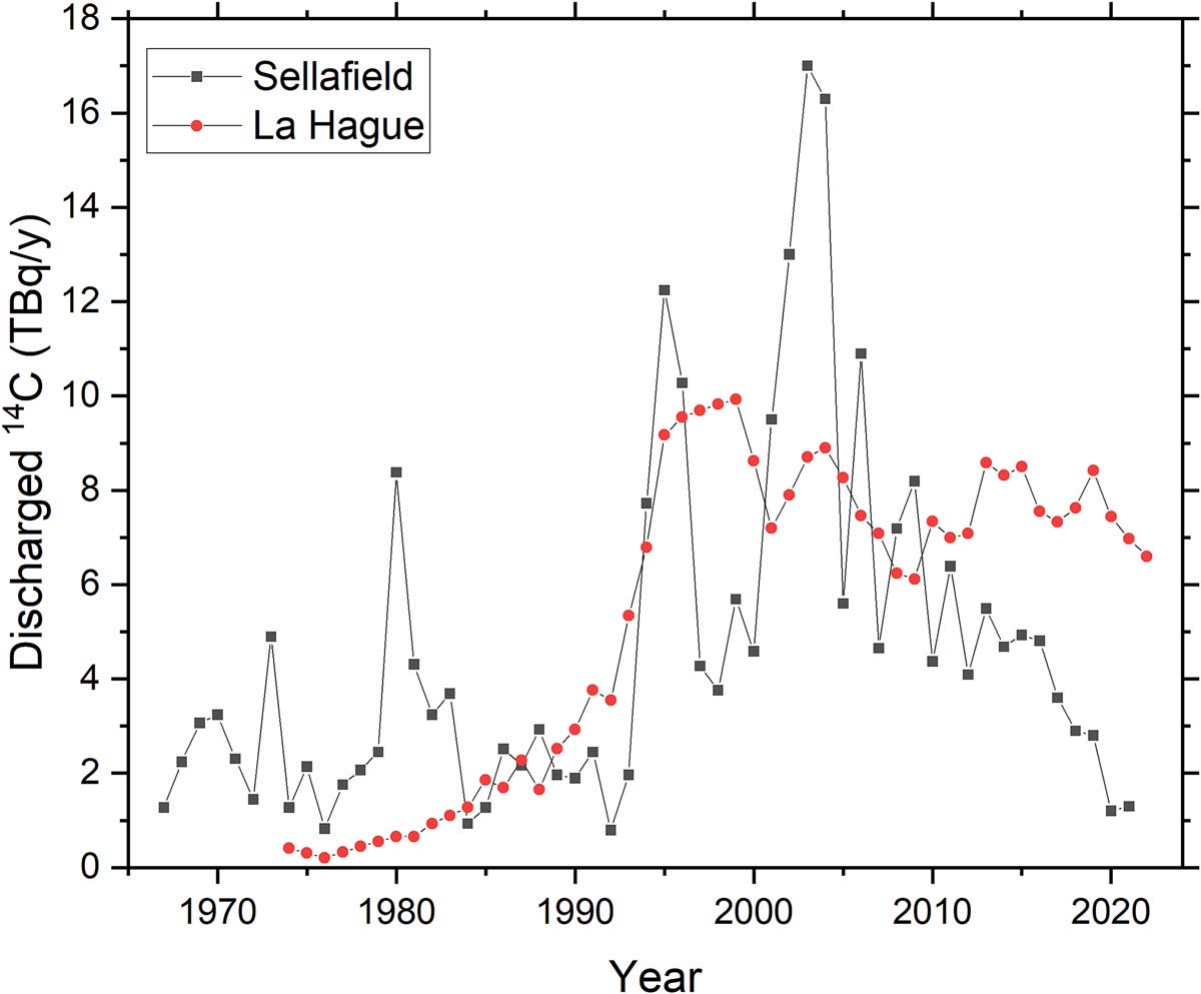
Liquid discharge rates of ^14^C for the reprocessing plants for spent nuclear fuel at La Hague (France) and Sellafield (UK) for 1967 to 2022 [19].

As can be seen in Fig. 1, the slopes of the atmospheric bomb pulse as well as of the average marine bomb pulse are nowadays very weak. It is therefore difficult to observe changes in F^14^C from 1 year to the next at a specific site, especially given analytical uncertainties and the many factors in the environment that can affect the ^14^C concentration (e.g. variations in precipitation and river runoff rates). However, Fig. 4 may still indicate some trends and patterns. For all sites in Skagerrak and Kattegat, F^14^C tends to decrease from 2020 to autumn 2022 or spring 2023, followed by an indication of a slight increase in autumn 2023. For 2024, all Kattegat and Skagerrak sites except Båteviken (Site 1), may indicate decreases in F^14^C. Variations in the annual liquid ^14^C discharges from La Hague and Sellafield may contribute to this pattern (Fig. 5). As we previously reported [6], other studies have estimated that ~10 per cent of the La Hague discharges reach Kattegat with a dilution factor of about 40, and a delay of 18 months or more [20]. For Sellafield discharges, the transport time has been estimated to 4–5 years, with the dilution factor being ~200 for the Kattegat waters (corresponding to that ~2 per cent of the Sellafield discharges reach Swedish coastal waters) [20–22]. Hence, the La Hague discharges are believed to dominate the excess ^14^C on the Swedish west coast, in particular since the discharges from Sellafield has been reduced in recent years (Fig. 5).

Due to the limited time period of the data in Fig. 4, as well as the small changes in F^14^C from year to year (overlapping with measurement uncertainties), this data set is not suitable for detailed correlation studies with emission data from La Hague. However, in a separate publication, we use the Särdal data series, covering the period 1967 to 2023, as basis for such discussions [15], showing that dramatic increases of discharged ^14^C from La Hague and Sellafield in the beginning of the 1990s reach Särdal a few years later [15].

Influence from Ringhals NPP on the F^14^C values in *Fucus* spp. for the sites in Fig. 4 (all located >10 km from the NPP) cannot be excluded, but is probably considerably less than the La Hague contribution (see below).

### Trends at Ringhals nuclear power plant

**Figure 6:**
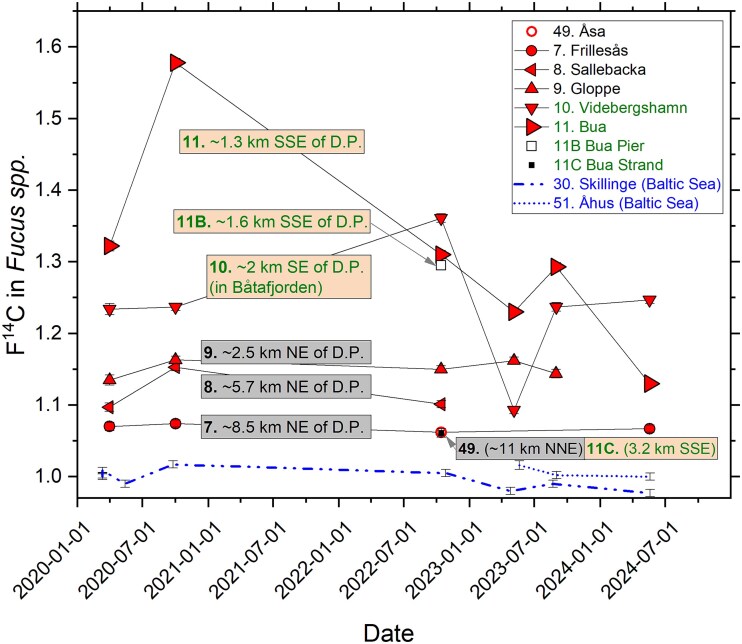
Temporal variation in F^14^C at sites close to Ringhals NPP, and at two reference sites in the Baltic Sea (sites 30 and 51). D.P.: discharge point of cooling water. Sites 49, 7, 8 and 9 are located north of the discharge point. Sites 10, 11B and 11C are located south of the discharge point. The uncertainty bars represent 1*σ*, resulting from repeated measurement of the same sample.

The temporal and spatial variations of F^14^C in *Fucus* spp. are pronounced at the sites located within 10 km of Ringhals NPP, as shown in Fig. 6 (also including F^14^C in *F. vesiculosus* from the two Baltic Sea reference sites). Site 11 Bua, located at Cape Krogstad at ~1.3 km SSE of the cooling water outlet (southern outlet direction), displays the highest F^14^C values for 4 of 6 sampling campaigns (up to F^14^C = 1.578 ± 0.006 in October 2020). This is ~50 per cent higher than sites far from the NPP (e.g. Site 4 Stockevik, ~85 km north-north-west, NNW, of Ringhals NPP, see Fig. 4). Site 10 Videbergshamn (at Cape Ringhals in the fjord Båtafjorden), also located south of the cooling water outlet, has the highest F^14^C for 1 of the 6 sampling campaigns (maximum F^14^C = 1.361 ± 0.006 in October 2022; about 30 per cent higher than Site 4 Stockevik, having F^14^C = 1.048 ± 0.006).

F^14^C in the *Fucus* spp. samples collected in October 2022 at two nearby Sites 11 (Bua) and 11B (Bua pier, ~200 m east of Site 11), both in fjord Båtafjorden, overlap within 2*σ* (F^14^C = 1.310 ± 0.006 and F^14^C = 1.295 ± 0.006, respectively). Site 11C, on the other hand, located further south at Cape Krogstad and facing Kattegat, shows a F^14^C in *Fucus* (F^14^C = 1.061 ± 0.006) that is not different from Site 4 Stockevik (~85 km NNW of Ringhals NPP, F^14^C = 1.048 ± 0.006; the data overlap within 2*σ*).

Ringhals Gloppe (Site 9), located close to the cooling water inlets of Ringhals NPP, has the third highest F^14^C values of the sites investigated (see the trends in Fig. 6). The distance from the cooling water outlet to Site 9 is about 2.5 km along the coastline in the northern direction. The surface water currents with main direction towards north and the whirlpools north of Cape Ringhals, as described by Notter [16], are considered responsible for the transportation of ^14^C from the cooling water outlet channels to the Gloppe site (Site 9). This is supported by the observation that the fourth highest F^14^C was found at Site 8 (Sallebacka), located ~5.7 km along the coastline from the cooling water outlet. At Sites 7 (Frillesås, ~8.5 km NE of the cooling water outlet) and 49 (Åsa, ~11 km NNE of the cooling water outlet), F^14^C overlaps within 2*σ* with the data from Site 4 Stockevik (~85 km NNW of Ringhals NPP) and from Site 46 Lysekil (~120 km north of Ringhals NPP).

**Figure 7:**
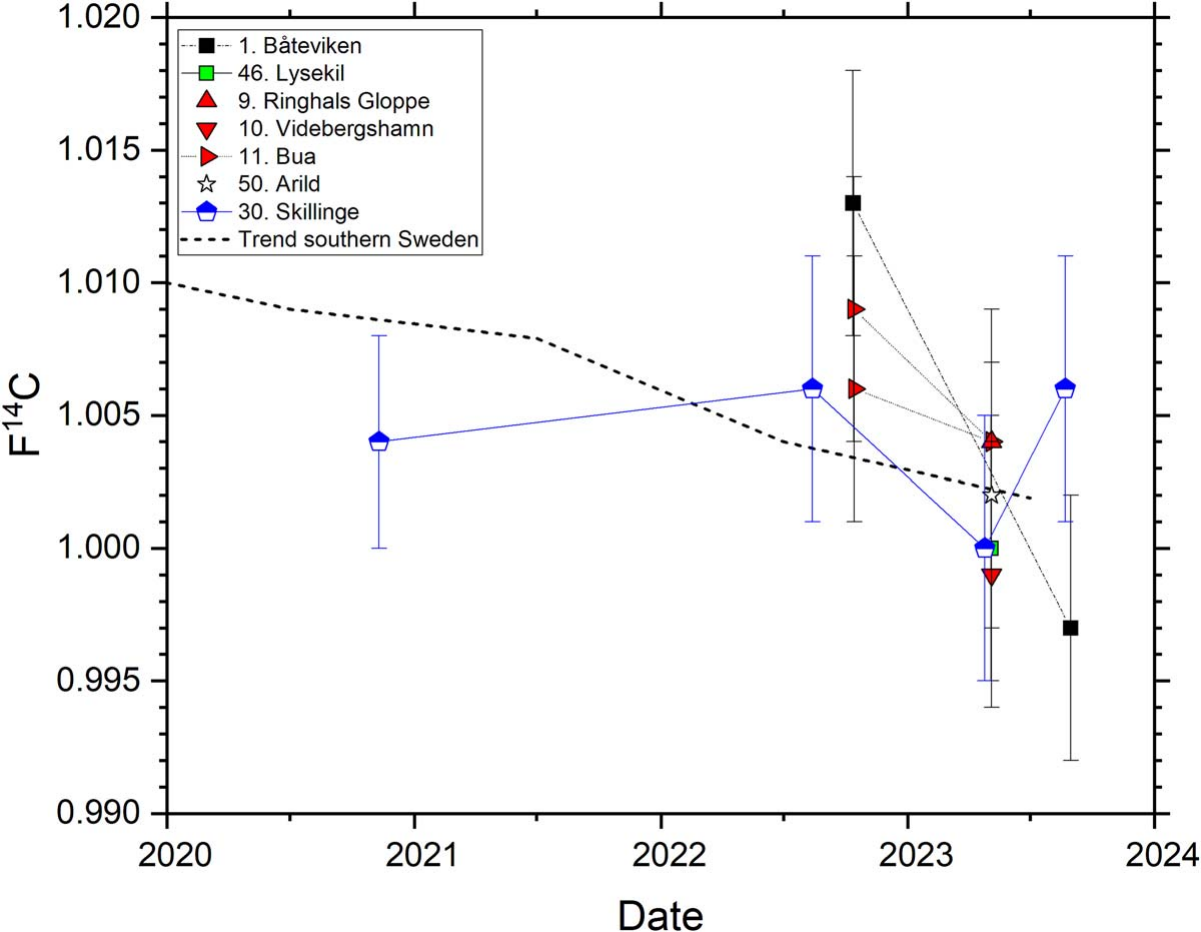
F^14^C in grass samples collected in the terrestrial environment at some of the marine sites. A trend line constructed from average F^14^C in vegetation in southern Sweden is also shown [25–27]. The standard deviation of F^14^C for each annual data set behind the trend line is small (0.004 or less) and the standard uncertainty of each annual mean is <0.001 [25–27]. The uncertainty bars of F^14^C in the grass samples represent 1*σ* (relative uncertainty <0.6 per cent), resulting from repeated measurement of the same sample.

In our previous study [6], no statistically significant differences in F^14^C were observed between *F. vesiculosus*, *F. serratus*, and *F. spiralis* for samples collected at Särdal (Site 15). However, as visualized in Fig. 3, there are differences in F^14^C for *F. vesiculosus* and *F. serratus* for sites close to Ringhals NPP for samples collected at Site 11 (Bua) in autumn 2020. At this point in the study, we used only the upper parts of the seaweed (from at least three plants), aiming to represent the *Fucus* tissue formed since last spring (vesicles and the parts above for *F. vesiculosus* and the upper 10 cm of *F. serratus*). For the samples collected in autumn 2020 at Bua (Site 11), the upper 10 cm of *F. serratus* had F^14^C = 1.367 ± 0.006, whilst *F. vesiculosus* (vesicles and above) had F^14^C = 1.578 ± 0.006 (i.e. 15 per cent higher than in *F. serratus*, see Supplementary Materials). Furthermore, for Gloppe (Site 10) north of Ringhals NPP, whole plants of *F. serratus* collected in spring 2023 had F^14^C = 1.121 ± 0.005, whilst *F. vesiculosus* (vesicles and above) had F^14^C = 1.162 ± 0.005 (i.e. 4 per cent higher than in whole plants of *F. serratus*). The differences in F^14^C between the two types of *Fucus* is most likely due to variable F^14^C in DIC in seawater close to Ringhals NPP in combination with differences in growing patterns and possibly in turnover of carbon. The biological half-life of ^14^C in *Fucus* from the Swedish west coast is to the best of our knowledge not well known. Fiévet et al. [23] estimate the biological half-life of ^14^C in macroalgae in the English Channel to 45 days. Keogh et al. [24] estimate a mean availability time of about 4 months for ^14^C in *Fucus* on the east coast of Ireland. None of these studies discuss possible differences between *Fucus* species.

In our previous study [6], we did not observe any statistically significant differences in F^14^C in various parts of *F. vesiculosus* individuals representing different years/periods of growth for samples collected in spring 2020, for example at Särdal (Site 15) and at the Baltic Sea reference site Skillinge (Site 30). However, for one relatively old individual of *F. serratus* (estimated to be several years of age with a total length of ~50 cm) collected in spring 2023 at Bua (Site 11) close to Ringhals NPP, the upper 40 cm had F^14^C = 1.235 ± 0.005, whilst the lower 10 cm (the older part) had F^14^C = 1.345 ± 0.006 (i.e. 9 per cent higher F^14^C than the upper parts). These observations may be due to higher growth rates in the upper parts of the *Fucus* plant than in the lower parts in combination with fluctuating F^14^C in DIC in seawater due to liquid discharges from Ringhals NPP.

### Terrestrial environment

The F^14^C data for grass sampled in the terrestrial environment of the marine sampling sites are summarized in Fig. 7, including a trend line of F^14^C in vegetation in southernmost Sweden. The trend line has been obtained from analysis of >10 vegetation samples per year and coincides with central European data [25–27]. The standard deviation of F^14^C for each annual data set behind the trend line is small (0.004 or less) and the standard uncertainty of each annual mean is <0.001 [25–27].

Most grass samples (Fig. 7) overlap within 1*σ* with the trend line for southern Sweden, and all data overlap within 3*σ*. No elevated F^14^C is observed in grass collected at the sites close to Ringhals NPP (9 Gloppe, 10 Videbergshamn, and 11 Bua in Fig. 7). These sites are however not in the main downwind direction. The absence of excess ^14^C in the terrestrial environment may also be due to the fact that the two operational reactors are PWRs, which are known do mainly emit ^14^C as hydrocarbons (not accessible to vegetation by photosynthesis), and to a lesser extent as CO_2_.

## Conclusion

*Fucus* spp. has been collected in shallow waters at the coastal line on the Swedish west coast in the period autumn 2020 to spring 2024. In general, F^14^C in *Fucus* on the Swedish west coast is higher than in *Fucus* from reference sites in the Baltic Sea, confirming the observation from a study from spring 2020 [6]. Our previous finding, from spring 2020, that F^14^C increases towards the north of the Swedish west coast is confirmed also for autumn 2020 and for 2022–24. As described above, liquid ^14^C discharges, mainly from spent nuclear fuel reprocessing plant at La Hague (France), are most likely responsible for the majority of the ^14^C on the Swedish west coast (excluding ^14^C of natural origin and from the atmospheric testing of nuclear weapons in the past).

In the immediate vicinity of Ringhals NPP, F^14^C in *Fucus* spp. is up to 50 per cent higher than sites located in what could be considered as reference areas (without impact from the NPP). The study indicates that such areas should be further away than ~8 km north (Site 7 Frillesås) and ~3 km south (Site 11C Bua strand), since no excess in F^14^C is observed compared to nearby sites located further away from the NPP. *Fucus* spp. growing close to the shore in the immediate marine vicinity of Ringhals NPP shows a high spatial as well as temporal variability in F^14^C. Fluctuations in ^14^C discharge rates from the NPP as well as changing dispersion patterns due to variations in water currents may contribute to the observed variabilities in *Fucus* F^14^C. We believe that these factors are responsible for the observation that F^14^C may vary between different parts (of different age) of individual *Fucus* plants from Ringhals NPP waters. Hence, to obtain the best conditions for comparative studies, future investigations should aim for using one *Fucus* species only with several individuals of the approximate same age per site.

The study has shown that Swedish west-coastal waters display a high spatial variation in F^14^C, whereas vegetation in the terrestrial environment does not (in this case, grass collected at some of the sites).

This study aimed to investigate the spatial and temporal variations in ^14^C at the Swedish west coast and is limited to the case of shallow waters close to land. Since *Fucus* spp. absorbs DIC from the surrounding seawater, other biogeochemical fractions containing ^14^C are not covered by the study. A more comprehensive radioecological investigation of ^14^C in the marine environment of Ringhals NPP is currently underway.

## Supplementary Material

Supplementary_Material_S1_ncaf032

Supplementary_Material_S2_ncaf032

Supplementary_Material_S3_ncaf032
